# Access, interest and equity considerations for virtual global health activities during the COVID-19 pandemic: a cross-sectional study

**DOI:** 10.1186/s41256-023-00333-y

**Published:** 2024-02-06

**Authors:** Lisa Umphrey, Alyssa Beck, Shuo Zhou, Enid Kawala Kagoya, George Paasi, Alexandra Coria, Jessica Evert, Marina Haque, Amy Rule, Molly M. Lamb

**Affiliations:** 1 Department of Pediatrics, University of Colorado School of Medicine, 13123 E 16th Ave, B302, Aurora, CO 80045 USA; 2 Center for Global Health, Colorado School of Public Health, 13199 E Montview Blvd, Ste 310, A090, Aurora, CO 80045 USA; 3 Department of Epidemiology, Colorado School of Public Health, 13199 E Montview Blvd, Ste 310, A090, Aurora, CO 80045 USA; 4 Department of Communication Studies, School of Communication and System Health Lab, Hong Kong Baptist University, No. 5 Hereford Rd, Kowloon, Hong Kong; 5 Department of Community Health, Institute of Public Health, Busitema University, P.O Box 1460, Mbale, Uganda; 6 Mbale Clinical Research Institute, Plot 29, 33 Pallisa, Mbale, Uganda; 7 Department of Pediatrics, Maimonides Children’s Hospital and SUNY Downstate College of Medicine, 4802 10th Ave, Brooklyn, NY 11219 USA; 8 Child Family Health International, 11135 San Pablo Ave #929, El Cerrito, CA 94530 USA; 9 Department of Anesthesiology, Wayne State University, Detroit, MI 48202 USA; 10 Emory University School of Medicine, 2015 Uppergate Dr, Atlanta, GA 30307 USA; 11 Children’s Healthcare of Atlanta, 2015 Uppergate Dr, Atlanta, GA 30307 USA

**Keywords:** Global health, Virtual, Pandemic, Education, Equity

## Abstract

**Background:**

Global health activities (GHAs) reduce health disparities by promoting medical education, professional development, and resource sharing between high- and low- to middle-income countries (HICs and LMICs). Virtual global health activities facilitated continuity and bidirectionality in global health during the COVID-19 pandemic. While virtual engagement holds potential for promoting equity within partnerships, research on equitable access to and interest in virtual global health activities is limited.

**Methods:**

We conducted a cross-sectional, online, mixed-methods survey from January to February 2022 examining access to virtual activities before and during the pandemic across resource settings. Eligible participants were participants or facilitators of global health activities. Closed- and open-ended questions elicited participants’ access to and interest in virtual global health engagement.

**Results:**

We analyzed 265 surveys from respondents in 45 countries (43.0% LMIC vs. HIC 57.0%). HIC respondents tended to report greater loss of in-person access due to the pandemic at their own institutions (16 of 17 queried GHAs), while LMIC respondents tended to report greater loss of in-person activities at another institution (9 of 17 queried GHAs). Respondents from LMICs were more likely to gain virtual access through another organization for all 17 queried VGHAs. HIC respondents had significantly more access to global health funding through their own organization (*p* < 0.01) and more flexibility for using funds. There were significant differences and trends between respondent groups in different resource environments in terms of accessibility to and interest in different virtual global health activities, both during and after the pandemic.

**Conclusions:**

Our results highlight the need to examine accessibility to virtual global health activities within partnerships between high- and low- to middle-income countries. While virtual activities may bridge existing gaps in global health education and partnerships, further study on priorities and agenda setting for such initiatives, with special attention to power dynamics and structural barriers, are necessary to ensure meaningful virtual global health engagement moving forward.

**Supplementary Information:**

The online version contains supplementary material available at 10.1186/s41256-023-00333-y.

## Introduction

Global Health (GH) focuses on advancing global and interdisciplinary healthcare [1–6], with special consideration of health inequities and the needs of underserved populations [7–9]. Global health activities (GHAs) focus on healthcare, capacity building, access to resources and technical expertise, professional mentorship, and collaborative education, research, or public health initiatives [2, 10–15]. GHAs often involve international travel among partner sites, such as for research or training activities. To promote equity in experience and opportunity, bidirectional visits of all partners to all sites is ideal [10, 14–19]. However due to inequitable immigration policies and lack of funding for health professionals from LMICs, it is often the professionals from HICs who travel to LMICs [16]. Global health (GH) partners in HICs therefore disproportionately travel to LMIC partner sites without reciprocity, which can exacerbate inherently inequitable power differentials affecting the discourse around GH and underserved populations [11, 15, 18, 20–22]. Over the last decade, research, training and educational activities have begun to utilize virtual platforms to promote bidirectional flows of knowledge sharing and physical visits [23], but inequities persist.

Strict infection control practices and unprecedented travel restrictions during the COVID-19 pandemic disrupted in-person GHAs and bidirectional activities [11, 24–27], and overall communication between GH partners, typically facilitated and reinvigorated by in-person site visits, likewise declined but in a disparate fashion for those is LMIC versus HIC settings [11, 27, 28]. Meanwhile GH inequities worsened at a time when knowledge sharing in the form of GHAs was most needed between all regions of the world, regardless of baseline resource levels, to acquire expertise in addressing pandemic-related healthcare and educational issues [26, 29]. Even as travel restrictions subside, shifting testing and vaccination requirements and ongoing funding cuts have continued to limit in-person GHAs in a post-COVID world, especially for those in LMIC settings [11, 25, 28, 30].

Due to pandemic-related travel challenges, GHAs conducted virtually online (VGHAs) became a critical element of GH collaboration and have tremendous potential to address challenges to GH engagement and equity. While there is substantial literature describing GH trainee competencies, preparation activities for short-term experiences in GH in resource-constrained settings, and sustaining GHAs during periods of disrupted in-person activities [9–11, 23, 24, 28, 31–34], few studies have explored successes and challenges to VGHAs from HIC and LMIC viewpoints. While the growing use of virtual platforms allowed for more participation in GHAs by those residing in LMICs, recent evidence documenting barriers, enablers, and preferences for VGHAs highlight the existence of new inequalities [23, 28]. Barriers to VGHA implementation included cost, lack of technological infrastructure, unilateral project ownership, and an absence of mutual learning and goal setting, and while challenges have been identified, solutions are still forthcoming.

While seeking to disrupt the status quo of inequitable and HIC-based GH practices, referred to as the decolonization of GH [20, 22, 35, 36], more research is needed to understand the benefits and detriments of VGHAs to build more equitable partnerships and to capitalize on an underutilized mode of bidirectional exchange. This mixed methods study aimed to follow up on preliminary descriptive data [28] by further characterising access to and interest in VGHAs by both LMIC and HIC partners before and during the COVID-19 pandemic. 

## Methods

### Study design

We conducted a cross-sectional, online, mixed-methods survey. The survey goals were to characterize changes in GHAs during the COVID-19 pandemic, measure perceived benefits of and barriers to VGHAs, and enrich our understanding of alternative strategies to maintain GHAs among participants and facilitators. We sought to understand how HIC versus LMIC designation affects access to and interest in virtual and in-person GHAs and materials both before and during the COVID-19 pandemic.

Our team developed initial survey questions as a tool to follow up on questions asked in a prior exploratory study [28] and to include conclusions from a systematic literature review [23] published by this author group. Using an interactive, consensus-based approach, we wrote 67 demographic and closed- and open-ended questions; we address qualitative portions of the survey in a separate manuscript. The survey and all study materials were available in English. Expanding on previous definitions and discussions of GH [1–6, 26, 37], we defined GHAs and related terminology in our survey according to Table 1.

**Table 1:** Global health study definitions

GHA and related terminology	Definition
Global Health Activity (GHA)	Any health activity focused on social accountability, equity, and cultural humility, which seeks to bridge geographical distance and/or resource levels. Activities are rooted in the collaborative, interdisciplinary practice of patient and population-centered healthcare and may focus on clinical, public health, research, community, policy, educational and/or development work. Further, activities may occur individually, between individuals, between organizations/institutions, or between individuals and organizations/institutions
Collaborative Global Health Activities	Activities in which members of all sides of a global health partnership(s) are involved in the preparation or presentation of the activity, and ideally members from both/several sides of a partnership attend and actively participate in the activity in real time
Global Health Experience or Elective	Engagement with global health electives, rotations, observer placements or other placements within or outside the organization’s catchment area
Global Health Participant	Any consumer of global health education materials or participant in global health activities, whether a student, post-graduate learner or adult learner pursuing continuing education
Global Health Facilitator	Any person who develops, facilitates, hosts, and/or provides global health education or activities to participants, either within one organization or within global health partnerships

The research questions guiding this analysis were as follows: (1) How does income designation (LMIC vs. HIC) affect type of access (virtual, in person, or both) to global health activities, both pre-pandemic and during the pandemic? (2) How does income designation (LMIC vs. HIC) affect interest in virtual global health activities, both during and after the pandemic?

Eligible participants were adult (> 18 years old) students, trainees or professionals in any discipline who participated in, created, taught, or facilitated GHAs as defined in Table 1, either independently or through an institution. Ineligible participants were those without direct engagement in GHAs.

### Data collection

The survey was open from 18 January to 14 February 2022. We targeted participants in the authors’ professional networks using a convenience sampling strategy, and we invited survey participation by email correspondence with 798 invitees. We sent reminder emails twice during the data collection period to encourage participation. We collected and stored data via the encrypted online survey platform, REDCap (Vanderbilt University) [38]. Participants received the REDCap survey link, a standard study information leaflet, and study contact details in an introductory email. Participants had to answer “yes” to three eligibility questions prior to beginning the survey. These included: (1) “Do you agree to participate in this survey? (2) “Are you at least 18 years old? and (3) “Are you involved in global health activities as defined above (including local or international clinical, public health, research, community, policy, educational and/or development activities)?” We de-identified all survey responses. Participants who fully completed the survey were eligible to enter a raffle to win an Amazon gift card.

Survey questions focused on participant engagement with GHAs before and/or during the pandemic (defined as before or after March 2020, to reflect when the most widespread lockdowns and travel restrictions worldwide began); whether this access was in-person or virtual; and whether the access was via the participant’s own or another organization, such as via a GH partner. The full survey is available in the Additional file 1: Appendix.

Based on our prior publications [23, 28, 39] and author group professional experience as GH educators, researchers, and clinicians, we grouped GHAs into broader categories that shared common elements. The category “Access to GHAs and resources” included access to professional resources that support GH activities, GH educational materials, GH didactic sessions, and GH simulation sessions. This category also include participation in the creation of GH materials. The category “GH experiences and electives” included, GH experience preparation sessions, hosting of GH participants from outside one’s own organization, and local, domestic/national, or international GH experiences. The category “Collaborative GHAs” referred to any GHAs involving both (or multiple) sides of a GH partnership, and included the following collaborative GH education sessions, clinical case support activities, research activities, and ward, clinical, or laboratory rounds. Finally, the category “Access to GH professional development” included access to GH mentorship, academic recognition for GH activities, and ability to participate in GH networking activities (such as conferences).

### Data processing and analysis

We calculated frequencies and percentages and used chi squared or Fisher’s exact tests to determine statistical significance for categorical comparisons. We categorized respondents’ country of residence as either an HIC or LMIC based on the World Bank 2022 fiscal year classifications [40] and used income status (HIC vs. LMIC) as the primary explanatory variable. We examined “Other” responses that included free text and recategorized based on study team judgement. We coded responses for access to GHAs as ‘1’ for being checked and ‘0’ for unchecked or missing, which allowed for a binary outcome. Respondents who marked “N/A” or “Don’t know” were coded as 0 for access to a GHA.

In order to assess additional factors influencing opinions about VGHAs, we ran a secondary analysis in which we classified the primary explanatory variable based on country of GH participation instead of country of residence. We performed all descriptive statistics with SAS (OnDemand, SAS Institute Inc.) and data presentation with R (v 1.4.1106, RStudio). We used an alpha of 0.05 to determine statistical significance.

### Ethical considerations

We obtained institutional review board approval from the Colorado Multiple Institutional Review Board (University of Colorado, Aurora, CO, USA; #21-3020).

## Results

We invited a total of 798 participants from the authors’ professional networks (such as departmental email groups) to complete the survey. A total of 347 respondents completed the eligibility questions, and of these, we removed 82 surveys for which the majority of questions were left blank. Thus, we retained 265 for analyses. A total of 154 participants provided adequate free-text responses for qualitative analysis, which will be addressed in a subsequent manuscript.

Survey response rate among known invitees was 43.5% (n = 347/798). We were informed that our survey also circulated on at least two large GH listservs, and we are unable to determine what percentage of subscribers to those sites responded to the survey.

## Demographic characteristics of survey respondents

**Fig. 1:**
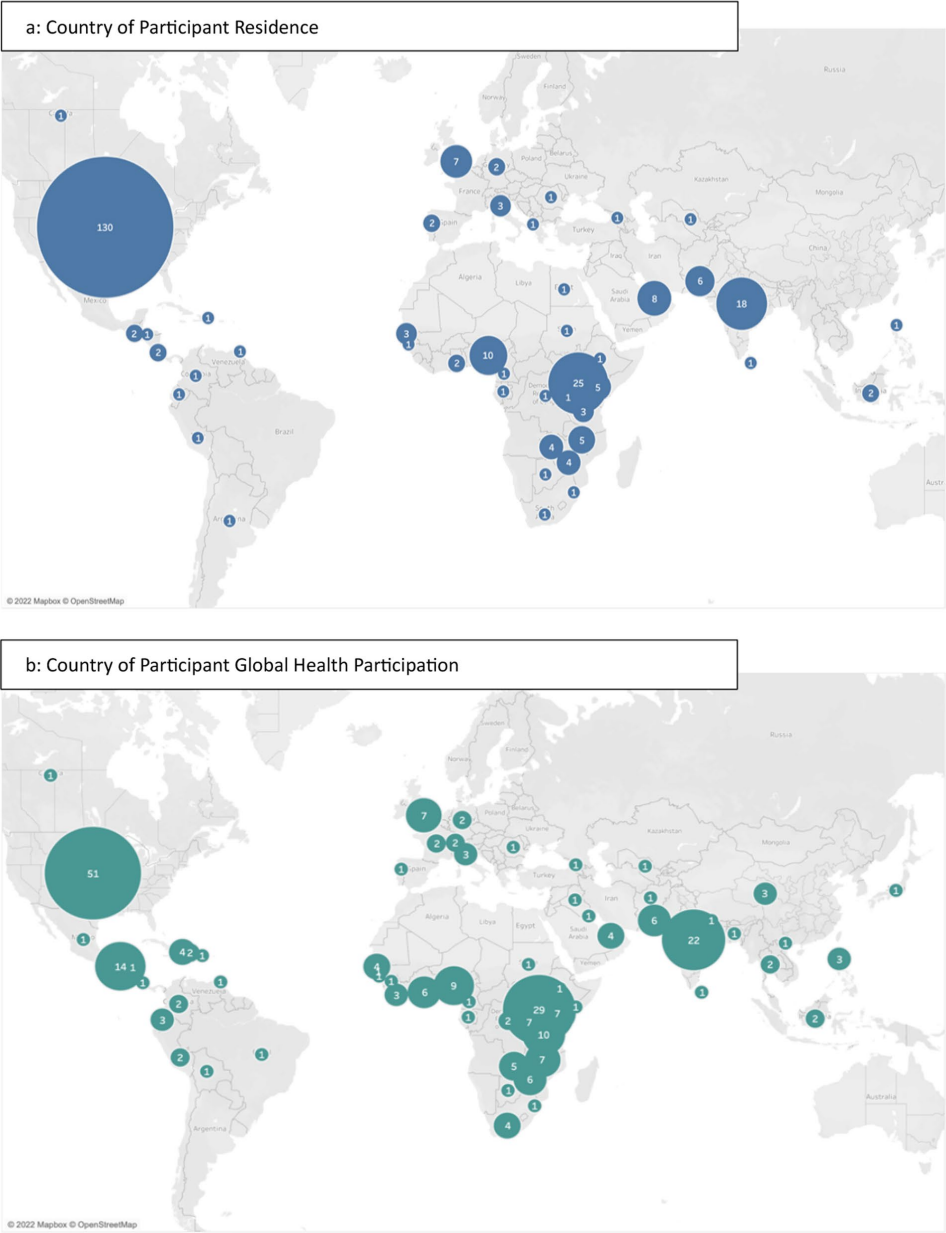
Participant country of residence (1a) and global health participation (1b)

See Table 2 for full demographic characteristics of survey respondents. Survey respondents reported 45 countries of current residence (Fig. 1a), the most common being the United States (n = 128, 48.3%), followed by Uganda (n = 25, 9.4%), India (n = 18, 6.8%), and Nigeria (n = 10, 3.8%). There were 151 respondents (57%) that resided in HICs versus 114 (43.0%) from LMICs (Table 2). The most reported primary country of GH participation was the United States (n = 49, 18.5%), followed by Uganda (n = 29, 10.9%), India (n = 22, 8.3%), Guatemala (n = 14, 5.3%), and Tanzania (n = 10, 3.8%) (Fig. 1b).

**Table 2:** Survey respondent demographic and global health engagement characteristics

Demographic characteristics	HIC	LMIC	Total
	n = 151	n = 114	n = 265
*Age*			
18–24.9 years	17 (35.4%)	31 (64.6%)	48 (18.1%)
25–34.9 years	49 (61.3%)	31 (38.8%)	80 (30.2%)
35–44.9 years	37 (56.1%)	29 (43.9%)	66 (24.9%)
45–54.9 years	18 (56.2%)	14 (43.8%)	32 (12.1%)
55–64.9 years	13 (72.2%)	5 (27.8%)	18 (6.8%)
65 years or older	16 (80.0%)	4 (20.0%)	20 (7.5%)
Prefer not to answer	1 (100%)	0 (0%)	1 (0.4%)
*Gender*			
Male	50 (45.9%)	59 (54.1%)	109 (41.1%)
Female	98 (64.1%)	55 (35.9%)	153 (57.7%)
Additional gender category	1 (100%)	0 (0%)	1 (0.4%)
Prefer not to answer	2 (100%)	0 (0%)	2 (0.8%)
*Education*			
Completed primary school	0 (0%)	1 (100%)	1 (0.4%)
Completed secondary school	1 (14.3%)	6 (85.7%)	7 (2.6%)
Some university	9 (29%)	22 (71%)	31 (11.7%)
Completed university	9 (24.3%)	28 (75.7%)	37 (14%)
Some post-graduate	22 (75.9%)	7 (24.1%)	29 (10.9%)
Completed post-graduate	110 (68.8%)	50 (31.2%)	160 (60.4%)
*Primary position*			
Academia/research	39 (57.4%)	29 (42.6%)	68 (25.7%)
Administrative	10 (83.3%)	2 (16.7%)	12 (4.5%)
Health professional	34 (58.6%)	24 (41.4%)	58 (21.9%)
Organizational leadership	3 (17.6%)	14 (82.4%)	17 (6.4%)
Public health/policy	8 (44.4%)	10 (55.6%)	18 (6.8%)
Trainee	57 (62%)	35 (38%)	92 (34.7%)
*Clinical specialty*			
Anesthesiology	3 (100%)	0 (0%)	3 (1.1%)
Dentistry	1 (33.3%)	2 (66.7%)	3 (1.1%)
Emergency medicine	15 (75%)	5 (25%)	20 (7.5%)
Family medicine	7 (31.8%)	15 (68.2%)	22 (8.3%)
Internal medicine	14 (70%)	6 (30%)	20 (7.5%)
Mental health	1 (50%)	1 (50%)	2 (0.8%)
Nursing	2 (16.7%)	10 (83.3%)	12 (4.5%)
Pediatrics	42 (87.5%)	6 (12.5%)	48 (18.1%)
Pharmacy	6 (31.6%)	13 (68.4%)	19 (7.2%)
Surgery	6 (60%)	4 (40%)	10 (3.8%)
Women’s health	3 (42.9%)	4 (57.1%)	7 (2.6%)
In training	7 (77.8%)	2 (22.2%)	9 (3.4%)
Other	5 (45.5%)	6 (54.5%)	11 (4.2%)
Non-clinician	39 (50%)	39 (50%)	78 (29.4%)
Unknown	0 (0%)	1 (100%)	1 (0.4%)
*Engagement type*			
Global health participant	44 (51.2%)	42 (48.8%)	86 (32.5%)
Global health facilitator	50 (66.7%)	25 (33.3%)	75 (28.3%)
Participant and facilitator	47 (56%)	37 (44%)	84 (31.7%)
Don’t know	3 (50%)	3 (50%)	6 (2.3%)
Missing	7 (50%)	7 (50%)	14 (5.3%)

*HIC* High-income country, *LMIC* Low and middle-income country

Respondents reported 17 primary languages of work or education, of which the most common were English (n = 229, 86.4%) Spanish (n = 10, 3.8%), and French (n = 9, 3.4%). The remaining 14 primary languages were reported by 2 respondents or less. Similar percentages of respondents identified as either GH participants, facilitators, or both.

## Funding and support for global health activities

**Fig. 2:**
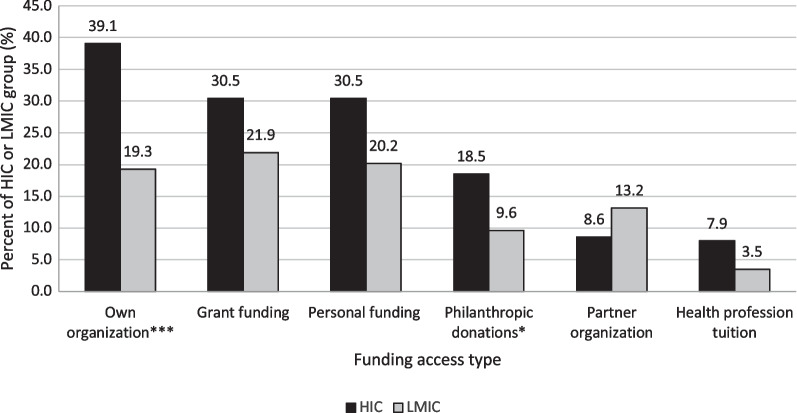
Access to various types of funding for global health activities according to survey respondents. Respondents could check all that apply to reflect personal access to each funding type. Responses are stratified by high-income country and low- to middle-income country based on country of residence of the survey respondent. Not all possible response options are shown. HIC = High-income country; LMIC = Low- to middle-income country; * p<0.05, ** p<0.01, *** p<0.001

Participants could describe multiple funding types applicable to their GHAs (Fig. 2a). HIC versus LMIC respondents reported overall more access to GH funding types, with a significant difference between GH funds provided via one's own organizational budget (n = 22/114, 19.3% LMIC; n = 59/151, 39.1% HIC, *p* < 0.01), and significantly more access to GH funding in addition to core role funding (n = 27/114, 23.7% LMIC; n = 50/151, 33.1% HIC, *p* = 0.02). LMIC respondents reported having no access to any GH funding significantly more often that HIC respondents (n = 41/114, 36.0% LMIC; n = 29/151, 19.2% HIC, *p* < 0.01) as well as having more access to funds via a partner organization (n = 15/114, 13.2% LMIC; n = 12/151, 8.0% HIC, *p* = 0.17).

Figure 2b shows permitted expenditures for GH funding among respondents. HIC respondents reported overall more flexibility in spending, while reporting significantly more use of GH funds towards GH travel compared to LMIC respondents (n = 22/63, 34.9% LMIC; n = 72/112, 64.3% HIC, *p* < 0.01).

Although not significant, during the COVID-19 pandemic, more LMIC versus HIC respondents reported decreased access to funding (n = 29/63, 46.0% LMIC; n = 38/112, 33.9% HIC; *p* = 0.08), while more HIC versus LMIC respondents reported no change to their GH funding (n = 16/63, 25.4% LMIC; n = 46/112, 41.1% HIC; *p* = 0.08). Only 14/175 (8.0%) respondents had better access to funding because of the pandemic (n = 7/63, 11.1% LMIC; n = 7/112, 6.3% HIC; *p* = 0.08). Nearly half of respondents indicated having access to administrative support for GH activities (n = 52/114, 45.6% LMIC; n = 79/151, 52.3% HIC; *p* = 0.28).

## Participant access to global health activities

Frequencies and percentages of GHAs are summarized in Table 3.

**Table 3:** Access or gain of access to virtual global health activities

	Loss of in-person GHA at own organization			Loss of in-person GHA at other organization			Gain of virtual access during pandemic through own organization			Gain of virtual access during pandemic through partner organization (keeping, leave here)		
	n (%)			n (%)			n (%)			n (%)		
	HIC (n = 151)	LMIC (n = 114)	*p* value	HIC (n = 151)	LMIC (n = 114)	*p* value	HIC (n = 151)	LMIC (n = 114)	*p* value	HIC (n = 151)	LMIC (n = 114)	*p* value
*Access to GH educational activities and resources*												
Professional resources supporting GHAs	37 (24.5)	27 (23.7)	0.8774	13 (8.6)	19 (16.7)	0.0463*	25 (16.6)	27 (23.7)	0.148	8 (5.3)	19 (16.7)	0.0025**
GH educational materials	34 (22.5)	23 (20.2)	0.6461	12 (8.0)	16 (14.0)	0.1104	25 (16.6)	30 (26.3)	0.0524	8 (5.3)	18 (15.8)	0.0045**
GH didactic sessions	56 (37.1)	31 (27.2)	0.0895	12 (8.0)	16 (14.0)	0.1104	52 (34.4)	31 (27.2)	0.2081	11 (7.3)	18 (15.8)	0.0281*
GH simulation sessions	33 (21.9)	20 (17.5)	0.3851	10 (6.6)	14 (12.3)	0.1121	30 (19.9)	25 (21.9)	0.6819	9 (6.0)	15 (13.2)	0.0432*
Participated in the creation of GH materials	30 (19.9)	18 (15.8)	0.3934	14 (9.3)	12 (10.5)	0.7339	27 (17.9)	23 (20.2)	0.6364	8 (5.3)	14 (12.3)	0.0414*
*GH experiences and electives*												
GH experience preparation sessions	38 (25.2)	25 (21.9)	0.5401	13 (8.6)	15 (13.2)	0.233	26 (17.2)	24 (21.1)	0.4296	8 (5.3)	12 (10.5)	0.1107
Local GH experiences	29 (19.2)	30 (26.3)	0.1683	14 (9.3)	15 (13.2)	0.3157	25 (16.6)	25 (21.9)	0.2683	11 (7.3)	14 (12.3)	0.1683
Domestic/national GH experiences	34 (22.5)	24 (21.1)	0.7754	13 (8.6)	16 (14.0)	0.1613	24 (15.9)	23 (20.2)	0.3663	9 (6.0)	14 (12.3)	0.0704
International GH experiences	48 (31.8)	18 (15.8)	0.0029**	20 (13.3)	12 (10.5)	0.5013	31 (20.5)	20 (17.5)	0.5416	12 (8.0)	14 (12.3)	0.2403
Hosting of GH participants from outside organization	34 (22.5)	22 (19.3)	0.5252	13 (8.6)	8 (7.0)	0.6348	16 (10.6)	15 (13.2)	0.5206	8 (5.3)	12 (10.5)	0.1107
*Collaborative GH activities*												
Collaborative GH education sessions	31 (20.5)	17 (14.9)	0.2398	21 (13.9)	12 (10.5)	0.4092	30 (19.9)	21 (18.4)	0.7674	14 (9.3)	11 (9.7)	0.9171
Collaborative ward, clinical, or laboratory rounds	21 (13.9)	12 (10.5)	0.4092	9 (6.0)	8 (7.0)	0.728	8 (5.3)	9 (7.9)	0.393	4 (2.7)	10 (8.8)	0.0485*
Collaborative clinical case support activities	18 (11.9)	17 (14.9)	0.4764	8 (5.3)	13 (11.4)	0.0685	13 (8.6)	18 (15.8)	0.0718	5 (3.3)	11 (9.7)	0.0320*
Collaborative research activities	25 (16.6)	15 (13.2)	0.4442	9 (6.0)	11 (9.7)	0.2604	16 (10.6)	21 (18.4)	0.0688	3 (2.0)	13 (11.4)	0.0028**
*Access to GH professional development*												
GH mentorship	27 (17.9)	19 (16.7)	0.7961	14 (9.3)	13 (11.4)	0.57	22 (14.6)	19 (16.7)	0.6402	9 (6.0)	13 (11.4)	0.1118
Academic recognition for GH activities	23 (15.2)	15 (13.2)	0.6334	11 (7.3)	8 (7.0)	0.9335	24 (15.9)	18 (15.8)	0.9816	7 (4.6)	9 (7.9)	0.2701
GH networking activities	42 (27.8)	22 (19.3)	0.1088	24 (15.9)	13 (11.4)	0.2964	36 (23.8)	22 (19.3)	0.3759	11 (7.3)	14 (12.3)	0.1683

*GH* Global health, *GHA* Global health activity, *HIC* High-income country, *LMIC* Low- to middle-income country

*p < 0.05, **p < 0.01, ***p < 0.001

### Overall access to VGHAs during the pandemic

**Fig. 3:**
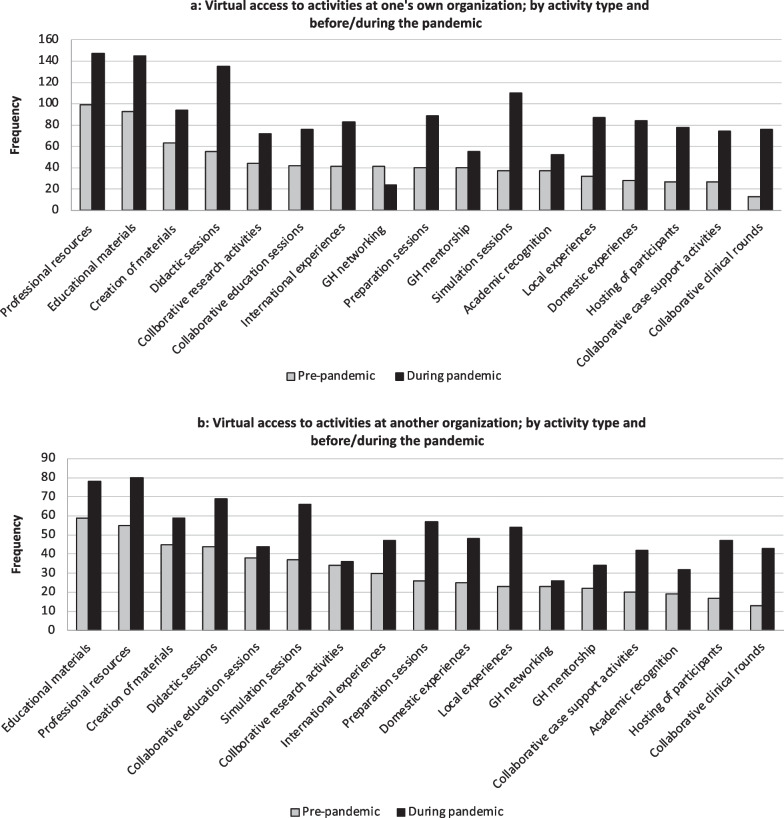
**A** Frequency of survey respondents that reported virtual access to various global health activities at their own organization. Responses are stratified by pre-pandemic (before March 2020) and during the pandemic (after March 2020). **B** Frequency of survey respondents that reported virtual access to various global health activities at another organization. Responses are stratified by pre-pandemic (before March 2020) and during the pandemic (after March 2020)

Across all activities, respondents reported an increase in virtual access to GHAs during the pandemic whether at their own or through another organization (Fig. 3a, b).

### Loss of in-person activities during the pandemic at one’s own organization

The most frequently lost in-person GHA at one’s own organization during the pandemic were GH didactic sessions (n = 31/114, 27.2%% LMIC; n = 56/151, 37.1% HIC, *p* = 0.09). There was a significant difference in loss of international GH experiences for HIC versus LMIC respondents (n = 18/114, 15.8%% LMIC; n = 48/151, 31.8% HIC, *p* < 0.01). Although most results weren’t significant, HIC respondents reported more loss of in-person access to 16 of 17 queried GHAs at their own institution than LMIC respondents.

### Loss of in-person activities during the pandemic at another organization

The most frequently lost in-person GHAs at another organization during the pandemic were GH networking (n = 13/114, 11.4%% LMIC; n = 24/151, 15.9%% HIC, *p* = 0.29) and collaborative GH education sessions with GH partners (n = 12/114, 10.5% LMIC; n = 21/151, 13.9% HIC, *p* = 0.41). Although most results weren’t significant, LMIC versus HIC respondents reported more loss of in-person access to 9 of 17 queried GHAs at another institution.

### Gain of virtual access during the pandemic through one’s own organization

The most gained VGHAs at one’s own organization during the pandemic were didactic sessions (n = 31/114, 27.2% LMIC; n = 52/151, 34.4% HIC, *p* = 0.21) and GH networking activities (n = 22/114, 19.3% LMIC; n = 36/151, 23.8% HIC, *p* = 0.38). Although most results weren’t significant, LMIC versus HIC respondents reported more gain of access to 12 of 17 queried VGHAs at their own institution.

### Gain of virtual access during the pandemic through another organization

Another subset of participants gained access to VGHAs through a partner organization during the pandemic as compared to one’s own organization. The most gained VGHAs at another organization during the pandemic were didactic sessions (n = 18/114, 15.8% LMIC; n = 11/151, 7.3% HIC, *p* = 0.03) and access to online professional resources (n = 19/114, 16.7% LMIC; n = 8/151, 5.3% HIC, *p* < 0.01). Although most results weren’t significant, LMIC versus HIC respondents reported more gain of access to all 17 queried VGHAs at another institution.

### Interest in virtual global health activities during and after the pandemic

**Fig. 4:**
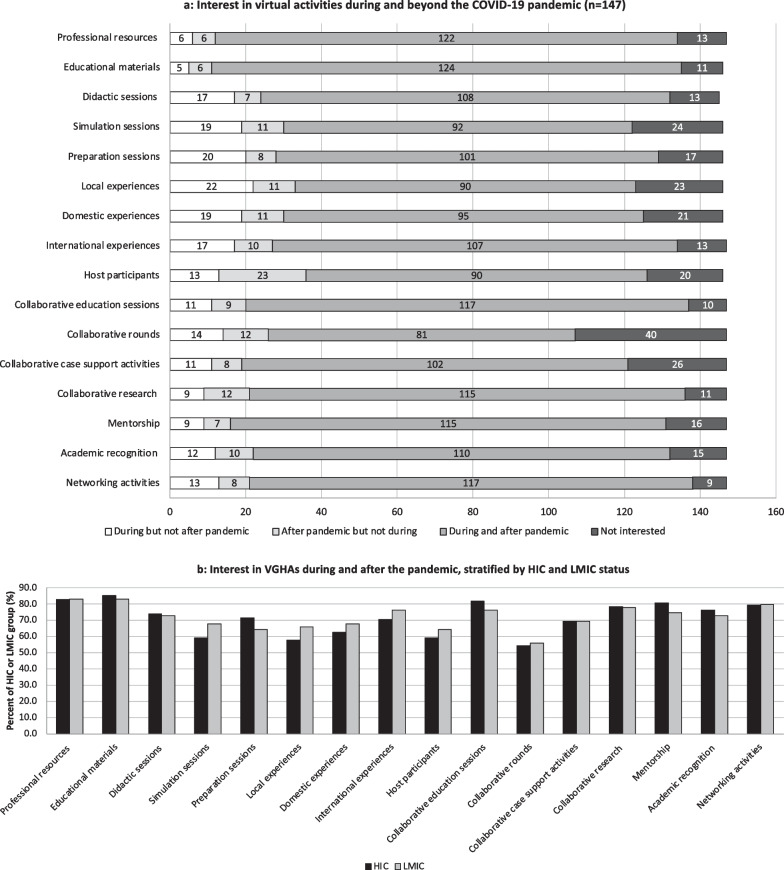
Interest in virtual global health activities during and after the pandemic

Among respondents who reported interest in VGHAs (n = 147/265, 55.5%; n = 59/114, 51.8% LMIC; n = 88/151, 58.3% HIC, *p* = 0.09), the majority expressed interest that began during but would continue after the pandemic (Fig. 4a). Among these respondents, few (n = 33/147, 22.5%) indicated interest in all possible VGHAs during and after the pandemic, which did not differ by HIC or LMIC status (*p* = 0.27). Respondents who reported interest that began during, but would continue after, the pandemic are stratified by HIC and LMIC (Fig. 4b).

### Secondary analysis

We reclassified participants using primary country of GH participation instead of country of residence to assess any differences that emerged in VGHA participation. We found that among those participating in GHAs in HIC contexts (68/265, 25.7%), 35/68 (51.5%) reported that their activities were conducted in resource-constrained environments within the HIC. Interest in GH activities during and after the pandemic did not differ by HIC/LMIC when redefined by country of participation.

## Discussion

To our knowledge, our study is the first examination of access to and interest in VGHAs of GH participants and facilitators during the COVID-19 pandemic. Further, our survey queries respondents from both LMIC and HIC settings to document differences in experiences with VGHAs by country of residence. Our data advance previous discussions about mutual support between GH colleagues during crisis [12, 41] and thoughtfully addressing LMIC partner needs during the pandemic [27] while building upon baseline data initially documenting perceptions of and barriers and facilitators for virtual global health partnership activities [28]. Additionally, our findings add a real-world perspective to recent discussions about shifting GHA activities virtually [11, 24, 28, 42] and complementing and coordinating efforts between GH colleagues, a tenet of ethical GH practices [9].

### Comment on respondents

Respondents represented multiple types of institutions active within 45 countries of residence. Four countries (USA, Uganda, India, and Nigeria) represented the majority of respondents, which likely reflect the most common GH partnerships reflected within the authors’ anglophone professional networks. GH participants and facilitators are similar to previously described members of GH partnerships [10, 14–17, 19, 21, 28], and our results offer insights into VGHA considerations for similar participants.

Of note, a quarter of our respondents represented those engaged in GHAs within resource-constrained areas of HICs, “global local” pairings between LMIC/LMIC or HIC/HIC partners whose unique needs should be considered during implementation of VGHAs [43, 44]. There is a growing body of evidence about these types of partnerships in the literature [23], but papers focusing specifically on “glocal” activities and needs of engaged partners, particularly for virtual engagement, are lacking. Our data and previous papers [23, 28] suggest that future study into these unique partner types would fill a gap in the literature.

### Funding and support for GH activities

Our results show an unbalanced trend related to access to GH funding and administrative support within our dataset. HIC respondents reported having significantly more access to GH funding through their own organization; were significantly more likely to have GH funding in addition to their core role funding; and tended to have more access to grants, personal funds, philanthropic donations, health professional tuition, and dedicated GH administrative support through their institution. Other types of funding for GHAs more accessible to those in LMIC settings (such as crowdsourcing and funding from non-governmental organizations) were extremely infrequently reported among our respondents (n = 3/151, 2%). For those reporting GH funding, HIC respondents reported overall more flexibility for using funds for various GH activities and significantly higher use of funds for GH travel compared to LMIC respondents. Perhaps most concerning, LMIC respondents were significantly more likely to report no access to GH funding at all.

These data support that GH funding was not equitably used or available to GH participants, particularly in terms of travel, a known barrier to bidirectionality within GH partnerships [16, 19]. We do not, however, have further details on why these trends emerged, although we speculate that this reflects the norm in GH partnerships for unidirectionality of the HIC partner visiting the LMIC partners. Our findings suggest that support to participants in LMICs is an urgent need within GH partnership and a critical gap in GH equity, particularly during the pandemic which exacerbated the normative model. Further study elucidating partner preferences for allocation of GH funding within partnerships and the use of available GH funds for traditionally expensive in-person activities versus less expensive but infrastructure-heavy activities will be important for improved partnership equity and resource allocation moving forward. Future research on the nuances of funding availability, distribution, and use among various GH participants and facilitators would fill a gap in the literature.

### Overall access to VGHAs during the pandemic

While funding was much more accessible to people located in HICs, VGHA accessibility did not vary significantly between the groups; this is an argument for the potential in improving equity of educational resources and bidirectionality through VGHAs. There were important trends to note in our data, however, which serve as cautions for GH partners engaging virtually. First, although not significant, HIC versus LMIC respondents did report overall more access to virtual GHAs, including more access to virtual collaborative GH education sessions, GH experience preparation sessions, and hosting of external GH participants. Second, LMIC partners reported more access to VGHAs related to their training and academic endeavors, such as virtual GH simulation sessions, clinical rounds, case discussions, or research. While we do not know our respondent’s priorities for their VGHAs, this trend may reflect the priorities of the HIC partner, such as a wish to continue training and teaching LMIC partners or continuing joint research beneficial to HIC institutions. These findings together highlight a potential lack of professional support and agency for faculty from LMIC engaged in GHAs which may exacerbate present inequities in capacity building and professional support available to LMIC partners virtually.

### Loss of in-person activities during the pandemic at one’s own or another organization

The majority of participants reported loss of in-person GHAs at both their own and another institution during the pandemic. Unsurprisingly, there was a significant difference (HIC > LMIC respondents) in the loss of in-person international experiences, with an overall greater HIC reported loss of in-person activities at one’s own institution and a greater LMIC reported loss of activities at another organization. Further, hosting of external GH partnerships was one of the most frequently lost GHAs for all respondents. These data corroborate trends elsewhere in the literature that although bidirectional GH experiences are preferred, most activities remain unidirectional with HIC to LMIC visits [19]. These findings may also reflect regional and unequal variabilities in quarantine requirements, travel restrictions, and access to more widespread GH networks when the pandemic began. The disparities in access are important considerations when planning how partners might sustain partnerships and GH activities during future periods of restricted travel or global unrest [23, 28, 39, 41].

### Gain of virtual access during the pandemic through one’s own or another organization

Although there were no significant differences between HIC and LMIC respondents in terms of overall gain of access to VGHAs during the pandemic through one’s own organization, there were upward trends in our data. Overall, LMIC respondents reported more gain of access to VGHAs at their own organization, but those activities did not include more recognition for their GH work (responses split evenly between HIC and LMIC respondents) nor more reported GH networking opportunities. This may suggest a prioritization for HIC partners to continue GH educational activities virtually for LMIC audiences, while focusing less on virtual GH professional development activities for LMIC colleagues.

Via a gain of access to VGHAs through another organization, LMIC respondents reported significantly more gain of access to GH educational activities and resources and collaborative GHAs. This may reflect a partnership strength among participants, demonstrating either that HIC partners sought to extend virtual experiences to their LMIC partners, or perhaps that LMIC partners requested virtual access from HIC partners. LMIC partners also tended to gain more virtual domestic/local GH experiences through their own organization. Little exists in the literature about in-person or virtual local/domestic GH experiences between LMIC-LMIC partnerships, and our finding suggests that those in LMIC settings pursued a virtual shift of GHAs due to the COVID-19 pandemic’s effect on in-person activities. This is a gap in the literature and an area of future study.

Regarding gain of virtual international GH experiences during the pandemic, HIC respondents tended to report more gain of virtual international GH experiences through their own organization, while LMIC respondents reported more gain of this activity through a partner organization. Further, LMIC respondents reported more virtual hosting of GH participants during the pandemic, both at their own and a partner organization. Although not statistically significant, this trend highlights an important consideration in terms of resource use, equity, and capacity enhancement.

### Interest in VGHAs

Our data indicate a widespread interest both during and after the pandemic for most types of VGHAs queried. Interestingly, stratifying our participants by country of GH participation did not reveal any significant difference in interests for VGHAs during or after the pandemic. These findings, in addition to previous studies [23, 28, 39], however, do not delve deeper into the striking lack of equity in terms of access to opportunities and availability of funding support for GHAs in LMICs. Based on our data, we recommend that every GH partnership should frankly evaluate each partner’s interest in VGHAs—both in terms of the specific activities possible within a partnership as well as how power structures at play in the partnership affect the communication of interest and therefore the prioritization of activities. VGHAs, because of their unique ability to bring all voices to the table, should be discussed in every GH partnership and collaboration moving forward to facilitate more equitable activity selection, prioritization, and implementation plans. Further study on how best to facilitate these discussions and agenda setting for VGHAs is merited.

### Future considerations

Barriers and enablers to VGHAs must be considered when making recommendations based on our data. A lack of internet connectivity is a severe concern for GHAs [23, 45–47], and our previous study [28] found that LMIC partners reported less access to wireless internet, less trainee access to organization-owned hardware, poorer cellular phone service, and less access to physical spaces like meeting and simulation facilities. The success of virtual engagement will require considering the technological capacity of GH actors and advocating for communication infrastructure investments, access to libraries and resources, and appropriate scheduling of meetings to ensure LMIC partner participation [28, 48]. Further, funding otherwise earmarked for GH travel and in-person activities could feasibly be shifted toward improving connectivity and other professional capacity building targeted to LMIC partner-sites.

### Limitations

Our study had several limitations. First, because our survey reached at least two large GH listservs despite targeted sampling among distinct invitee groups, we must estimate our response rate. Second, to focus on augmenting previous baseline data and to not exclude respondents who may not have had access to VGHAs at the time of the survey, we did not query respondents about lessons learned from virtual engagement. Third, the survey length may have contributed to survey respondent fatigue and contributed to missing information. For example, the survey instructed respondents to use the “N/A” column for GHAs that they did not have access to. However, most respondents left activities blank instead of using the “N/A” column in the survey to indicate lack of access. For this reason, we coded responses for access to GHAs as ‘1’ for being checked and ‘0’ for unchecked or missing, “N/A” or “Don’t know.” This limits our ability to distinguish between true lack of access versus missing responses for individual GHAs. Fourth, our study was only available in English, likely contributing to a sampling bias. Fifth, because of the convenience sampling strategy, these results may not be generalizable beyond the study population. Last and most importantly, our categorization of participants into HIC versus LMIC groups for analysis has inherent practical and ethical implications [49, 50] that affect the interpretation of our results.

Despite the limitations, we believe our results deepen our understanding of previous baseline data on VGHAs during the COVID-19 pandemic, and our data create a stronger foundation for future study of the implementation of VGHAs more widely in the wake of the pandemic. This body of data is important to guide future study, to provide a “before” comparison to help other groups with similar goals evaluate the impact of the pandemic on their GHAs, and to foster meaningful discussion within GH partnerships related to resource access, agenda setting, and equity in decision making.

## Conclusions

Our results highlight the need to examine accessibility to GHAs within HIC-LMIC partnerships, and VGHAs may help to more equitably bring both sides of GH partnerships to the table. A reorientation toward VGHAs with a deeper understanding of the vast inequities that exist in GH and to support GH equity will necessitate refocusing on funding imbalances, accurately identifying the needs of each partner, and prioritizing communication infrastructure to ensure each partner can engage in equitable decision making, improving activity accessibility, and activity implementation. Further study on priorities and agenda setting for VGHAs within partnerships, with special attention to power dynamics and structural barriers at play, are necessary to ensure meaningful virtual GH engagement moving forward.

### Supplementary Information

**Additional file 1. Appendix.** Complete data collection tool, Virtual Global Health Activities (VGHA) International Survey Study.

## Data Availability

The datasets used and/or analyzed during the current study are available from the corresponding author on reasonable request.

## Abbreviations

GH: Global health
GHA: Global health activity
VGHA: Virtual global health activity
HIC: High-income country
LMIC: Low- to middle-income country
